# Toward Discovering New Anti-Cancer Agents Targeting Topoisomerase IIα: A Facile Screening Strategy Adaptable to High Throughput Platform

**DOI:** 10.1371/journal.pone.0097008

**Published:** 2014-05-08

**Authors:** Yu-Shih Lin, Wan-Chen Huang, Mei-Shya Chen, Tao-shih Hsieh

**Affiliations:** 1 Institute of Cellular and Organismic Biology, Academia Sinica, Taipei, Taiwan; 2 Department of Biochemistry, Duke University Medical Center, Durham, North Carolina, United States of America; University of Iowa, United States of America

## Abstract

Topoisomerases are a family of vital enzymes capable of resolving topological problems in DNA during various genetic processes. Topoisomerase poisons, blocking reunion of cleaved DNA strands and stabilizing enzyme-mediated DNA cleavage complex, are clinically important antineoplastic and anti-microbial agents. However, the rapid rise of drug resistance that impedes the therapeutic efficacy of these life-saving drugs makes the discovering of new lead compounds ever more urgent. We report here a facile high throughput screening system for agents targeting human topoisomerase IIα (Top2α). The assay is based on the measurement of fluorescence anisotropy of a 29 bp fluorophore-labeled oligonucleotide duplex. Since drug-stabilized Top2α-bound DNA has a higher anisotropy compared with free DNA, this assay can work if one can use a dissociating agent to specifically disrupt the enzyme/DNA binary complexes but not the drug-stabilized ternary complexes. Here we demonstrate that NaClO_4_, a chaotropic agent, serves a critical role in our screening method to differentiate the drug-stabilized enzyme/DNA complexes from those that are not. With this strategy we screened a chemical library of 100,000 compounds and obtained 54 positive hits. We characterized three of them on this list and demonstrated their effects on the Top2α–mediated reactions. Our results suggest that this new screening strategy can be useful in discovering additional candidates of anti-cancer agents.

## Introduction

DNA transactions that transmit and restore genetic information invariably involve unwinding and rewinding of double helical structures. Because of the topological linkage between secondary and higher order DNA structures, the helical unwinding/rewinding result in the entanglement and interlocking of chromosomes and DNA. These topological entanglements would have to be resolved in a timely and precise manner, without which cells cannot survive. Topoisomerases are nature's solution to these seemingly intractable topological complexities [1]–[6]. They carry out these topological transformations through a cycle of reversible transesterification between the active site tyrosyl residue and phosphate backbone in DNA. The transient enzyme/DNA adduct creates a DNA gate through which another DNA segment can be transported and results in topological changes. Based on the structure and mechanism, topoisomerases are classified into two types: type I enzyme mediates the strand transport through a single strand DNA gate while type II enzyme transports DNA segment through a double strand gate. Both types of topoisomerases are further classified into two families, A and B, and these enzymes are ubiquitous in nature and have essential functions for growth and development of all organisms [7], [8].

Interestingly, the transient and reversible DNA breaks mediated by topoisomerases, so critical for their biochemical and biological functions, also create an Achilles' heel for the cells. Many cytotoxic agents, man-made or nature-produced, target at this step of the topoisomerase reaction and stabilize the cleavage intermediate thus generating potentially lethal DNA strand breaks [5], [9]–[14]. Some of these topoisomerase-targeting agents have proven to be clinically important anti-cancer drugs and antibiotics. The therapeutic efficacy of these life-saving drugs is frequently compromised because of the rise of drug-resistance in tumor or microbial cells. Searching for new modalities and drugs becomes ever more urgent if we want to keep pace with the threat of drug resistance. Because of the essential roles of topoisomerases in cell proliferation and because they are proven targets of clinically useful drugs, it is reasonable to expect that intensive efforts should be devoted to discover more drugs targeting topoisomerases. However, the biochemical assays that are most often used for monitoring topoisomerase activities are not readily adaptable for automation and high throughput platform. For biochemical assays the most versatile assays are based on the use of agarose gel electrophoresis since it can detect DNA structural transitions associated with the strand cleavage and passage activities of topoisomerases. The labor-intensive nature and the cumbersome process in acquiring the data make gel electrophoresis unlikely a method of choice for automation and quantification.

To rectify this difficulty, there are a number of approaches developed recently that are adaptable for automated high throughput platform to identify topoisomerase-targeting compounds [15]–[18]. They are all fluorescence-based topoisomerase activity assays, thus more amenable for automated quantification. However they are also characterized with the use of a plasmid DNA in the process of assays. We report here our effort in the development of a new approach using a fluorophore-tagged oligonucleotide duplex as a substrate for assaying the formation of stabilized topoisomerase/DNA complexes in the presence of a specific candidate agent. We describe our initial screen using this assaying technique of a chemical library with 100,000 compounds and characterization of three of the positive hits. This screening method may provide an alternative approach to discover new topoisomerase-targeting agents and enrich our arsenal to combat tumors and microbial pathogens.

## Materials and Methods

### Purification of Human Top2α

We used an engineered YEpWob6, a vector with hexahistidine-tagged human Top2α under a *GAL1* inducible promoter, to express recombinant human Top2α in yeast [19]. Yeast cells after galactose induction were harvested, resuspended in hypertonic buffer (1 M sorbitol, 20 mM potassium phosphate pH 7.4, 20 mM 2-mercaptoethanol) and lysed by adding lytic enzyme (Zymolyase 100T, Amsbio). Nuclei were harvested by centrifugation (20 min at 55,000X*g*), resuspended in a buffer of 20 mM potassium phosphate pH 7.4, 0.2% Triton X-100, 5 mM 2-mercaptoethanol with a protease inhibitor cocktail including 1 mM PMSF, 2 µg/mL Leupeptin, and 1 µg/mL Pepstatin A, and homogenized. We extracted Top2α from nuclei by increasing the NaCl concentration to 500 mM and removed nuclear pellet by centrifugation at 15,000X*g* for 25 min. The supernatant was fractionated by 3 chromatographic steps (GE Healthcare). The first one was a HisTrap FF crude Ni-chelating column and Top2α was eluted at 40 mM imidazole with a gradient of 20–200 mM imidazole, in 20 mM potassium phosphate pH 7.4, 500 mM NaCl and 0.1% Triton X-100. The pooled fractions were diluted three times by 20 mM potassium phosphate pH 7.4, and further purified through HiTrap SP column. Top2α eluted at 500 mM NaCl in a gradient of 200–800 mM NaCl. The pooled fractions were diluted three times by 20 mM potassium phosphate pH 7.4, and loaded to the Mono S 5/50 GL column. Top2α was eluted at 500 mM NaCl and the fractions were pooled, dialyzed overnight against 50% glycerol, 15 mM sodium phosphate pH 7.5, 150 mM NaCl, 0.1 mM EDTA, 1mM PMSF and 5 mM 2-mercaptoethanol, and stored at −20°C. The final Top2α fraction is over 90% pure and has a specific activity of at least 10^6^ units/mg with one unit defined as the amount of enzyme necessary to relax 0.5 µg pUC19 DNA in 30 min.

### DNA Substrates

Fluorescent DNA substrate (Integrated DNA Technologies, Inc., Coralville, IA) was designed similar to what was described previously (Smiley, Collins et al. 2007), a 29-bp oligonucleotide duplex containing a Top2 cleavage site in the center and Alexa Fluor 488 at the 3′ end. Plasmid pUC19 DNA purified by CsCl double-banding, phenol-chloroform extraction and alcohol precipitation was used for relaxation and cleavage assays (Sander and Hsieh 1983).

### Pre-HTS (High Throughput Screening) Anisotropy Assay

In 50 µl of Top2 reaction buffer containing 10 mM Tris-HCl pH 7.9, 5 mM MgCl_2_, 50 mM NaCl, 0.1 mM EDTA, 0.5 mM ATP. 50 nM Alexa Fluor 488-labeled DNA, 1.25 µM human Top2α, and teniposide (VM26) in a range of concentrations from 60 to 300 µM, the reaction mixture was incubated at 37°C for 30 minutes. Before measuring the anisotropy, NaClO_4_ was added to give a final concentration of 250 mM. Experiments testing the effects of NaClO_4_ concentrations showed that within a range of 100–750 mM it has a similar effect in modulating fluorescence anisotropy (to be detailed in **Results** section).

To test the effects of order of addition, 50 nM Alexa Fluor 488-labeled DNA, 250 nM human Top2α, and 250 mM NaClO_4_ with or without 300 µM VM26 were introduced in different order to 50 µl reaction buffer. The overall reaction time was 30 min at 37°C, unless otherwise noted.

To examine the positive hits by anisotropy assay under bulk conditions, the reaction was performed in 50 µl of reaction buffer containing 50 nM Alexa Fluor 488-labeled DNA, 250 nM human Top2α, and 15 µM test compound. The reaction mixture was incubated at 37°C for 30 minutes before adding NaClO_4_.

Anisotropy assays under bulk conditions were measured by Fluorolog-3 Spectrofluorometer (HIROBA Scientific) equipped with two polarizers in the paths for excitation and emission using the L-format method. The excitation and emission wavelength were 492 nm and 515 nm, respectively, each with a slit width of 2 nm, and the gain G value set at 1.

### High Throughput Anisotropy Screening

For HTS, screenings were conducted at the Genomics Research Center of Academia Sinica in Taiwan, following the protocol published previously and using the chemical library generated and collected there [20]. Briefly, screenings were performed in 1536-well plates using the high-throughput screening (HTS) system manufactured by GNF systems (GNF, San Diego, CA). The HTS system is integrated with dispensers, a 1536-pin transferring tool, incubators and a ViewLux plate reader (Perkin Elmer Inc.). The reaction volume was 4 µl per well containing 100 nM Alexa Fluor 488-labeled DNA with 150 nM human Top2α in the reaction buffer. After 30 minutes incubation at 37°C, 100 mM NaClO_4_ was added to each well to stop the reaction and the anisotropy value was measured. The screening was conducted with a representative library of 100,000 chemicals at a concentration of 12.5 µM. 300 µM teniposide (VM26) was included as a positive control, whereas 5% DMSO was the negative control. Z was calculated using the following formulation: 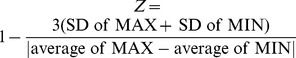. SD of MAX and SD of MIN are the standard deviation of positive and negative controls. A screening method suitable for HTS platform will have a Z factor greater than 0.5. With the first round screening of over 100,000 chemicals, 107 test compounds were scored positive by the criteria to be described in the text. Second round of screening was carried out by testing the anisotropy readouts at 8 different concentrations of each candidate compound using two-fold dilutions starting from 12.5 µM. Only the compounds resulting in 20% increases in detected signals over the negative controls, and showing dose-dependent responses at two or more points were defined as hits. There were 54 hits scored in the second round of screening.

### Relaxation Assays

In 20 µl of Top2 reaction buffer containing 8.5 nM negative supercoiled pUC19, 0.125 nM human Top2α with either 5% DMSO, or 60 µM test compounds, the reactions were performed at 37°C for 5, 10 or 15 minutes, terminated by adding SDS to 0.25%, EDTA to 10 mM and proteinase K to 0.4 mg/ml, further incubated at 50°C for 1 hour, and analyzed by electrophoresis in 1.2% agarose gel.

### Cleavage Assays

In a reaction buffer containing 8.5 nM negative supercoiled pUC19, 3.75 nM human Top2α and 150 µM teniposide (VM26) or as specified in the texts, the reactions were incubated at 37°C for 15 minutes, and terminated by adding SDS to 0.25%, EDTA to 10 mM and proteinase K to 0.4 mg/ml, or by adding NaClO_4_ to 500 mM for 2 minutes followed by addition of proteinase K to 0.4 mg/ml. Incubation was continued at 50°C for 1 hour and the samples were analyzed by electrophoresis in 1.2% agarose gel containing 0.15 µg/ml ethidium bromide. For testing the cleavage reactions with a linear DNA substrate, pUC19 was digested with ScaI to produce a unit length molecule. The restriction enzyme treated DNA was purified and used in the cleavage reactions.

To test the effects of positive hits in cleavage reaction, test compounds were diluted into three different concentrations, 16, 64 and 256 µM, and the reactions were carried out and analyzed as described above.

### Glycerol Gradient Sedimentation and Topoisomerase Detection by Immunoblots

A 3.5 ml glycerol gradient from 30–60% in Top2 reaction buffer was prepared in a polyallomer centrifugation tube, and layered with an 80 µl of Top2 reaction mixtures as described in texts. Centrifugation was carried out at 55,000 rpm for 16 hours at 20°C in TFT-80.4 Rotor (Thermo Scientific). A 22-gauge needle was used to pierce the tube and fractions were collected from the bottom. 50 µl sample of each fraction was loaded on a presoaked PVDF membrane using a slot blot manifold (Hoefer PR 648) apparatus. Following loading, the membrane was blocked for 1 hour at room temperature in 5% skimmed milk made in TBS buffer (25 mM Tris-HCl pH 8.0, 125 mM NaCl). Blocked membrane was incubated overnight at 4°C with a goat antibody against human Top2α (1∶5000; Santa Cruz Biotechnology, sc-5348). The membrane was then processed for chemi-luminescence with ECL reagent (Millipore, WBKLS0500) following manufacturer's recommendations.

### Cytotoxicity assay

Cytotoxicity assay was performed with HCT116 cells using the CellTiter 96 AQ_ueous_ One Solution Cell Proliferation Assay kit according to the manufacturer's protocol (Promega). Briefly, HCT116 cells were seeded in the 96-well plates at a cell density of 7500 cells per well in DMEM for 24 hour. After removing medium, exponentially growing cells were incubated with 1 µl of test compound at 6 two-fold dilutions in a final volume of 100 µl in each well. After a 72-hour incubation at 37°C, cells were replenished with fresh medium containing 10 µl MTS tetrazolium and incubated for one hour. The formazan converted by the live cells was monitored at 490 nm. Control cells contain 1% DMSO without any test compound. All experiments were repeated at least three times.

## Results

**Figure 1:**
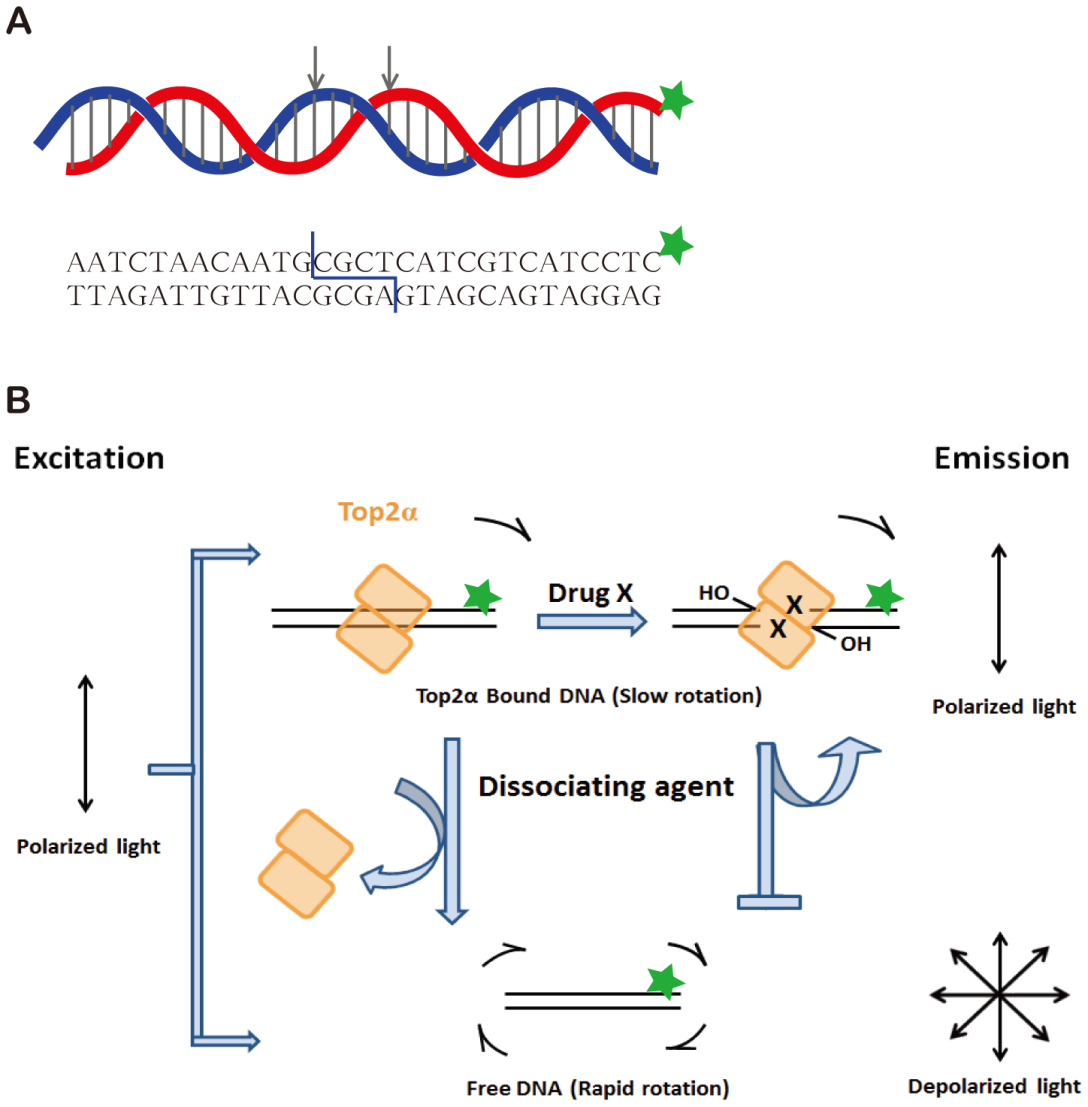
A schematic diagram of fluorescence-based screening for Top2α targeting compounds. (A) The fluorescent DNA substrate was synthesized with a known Top2 cleavage site (indicated by arrows). Alexa Fluor 488 marked by asterisk was labeled at the 3′end. (B) Schematic representation of the anisotropy-based Top2α drug screening. The topoisomerase-DNA complex, covalent or non-covalent, has a slower rotational diffusion and results in a higher anisotropy. After treated with a protein-dissociating agent, the non-covalent bound Top2α was removed, and the free DNA has a lower anisotropy. If a Top2α-targeting agent stabilized covalent enzyme-DNA complexes, the anisotropy will remain high.

We used purified full-length human Top2α and fluorophore-labeled, double-stranded oligonucleotide containing a topoisomerase binding site to develop the fluorescence-based assay (Fig 1A). The scheme of screening for Top2α-targeting compounds is illustrated diagrammatically in Fig 1B. This assay is based on the measurement of fluorescence polarizability (anisotropy) as an indication whether a protein is bound to fluorophore-tagged DNA. Upon excitation with polarized light, the emission from fluorophore of DNA substrate if staying immobile, is also polarized. Rotational diffusion changes the direction of the transition moment and causes depolarization. The topoisomerase-DNA complex, covalent or non-covalent, has a lower tumbling rate and results in a higher anisotropy. On the other hand, the free DNA has a higher tumbling rate and thus a lower anisotropy. Since anisotropy assay can be readily automated, we can carry out high-throughput screening (HTS) to identify potential lead compounds targeting against Top2α. However, one key challenge for this screening assay to work properly is to differentiate the drug-stabilized Top2α–DNA complexes from the bipartite Top2α-DNA complexes. We will need a reagent to disrupt the latter complex but maintain the former one.

### Treatment with NaClO_4_ can result in an increase in anisotropy dependent upon the presence of an anti-cancer drug teniposide (VM26)

**Figure 2:**
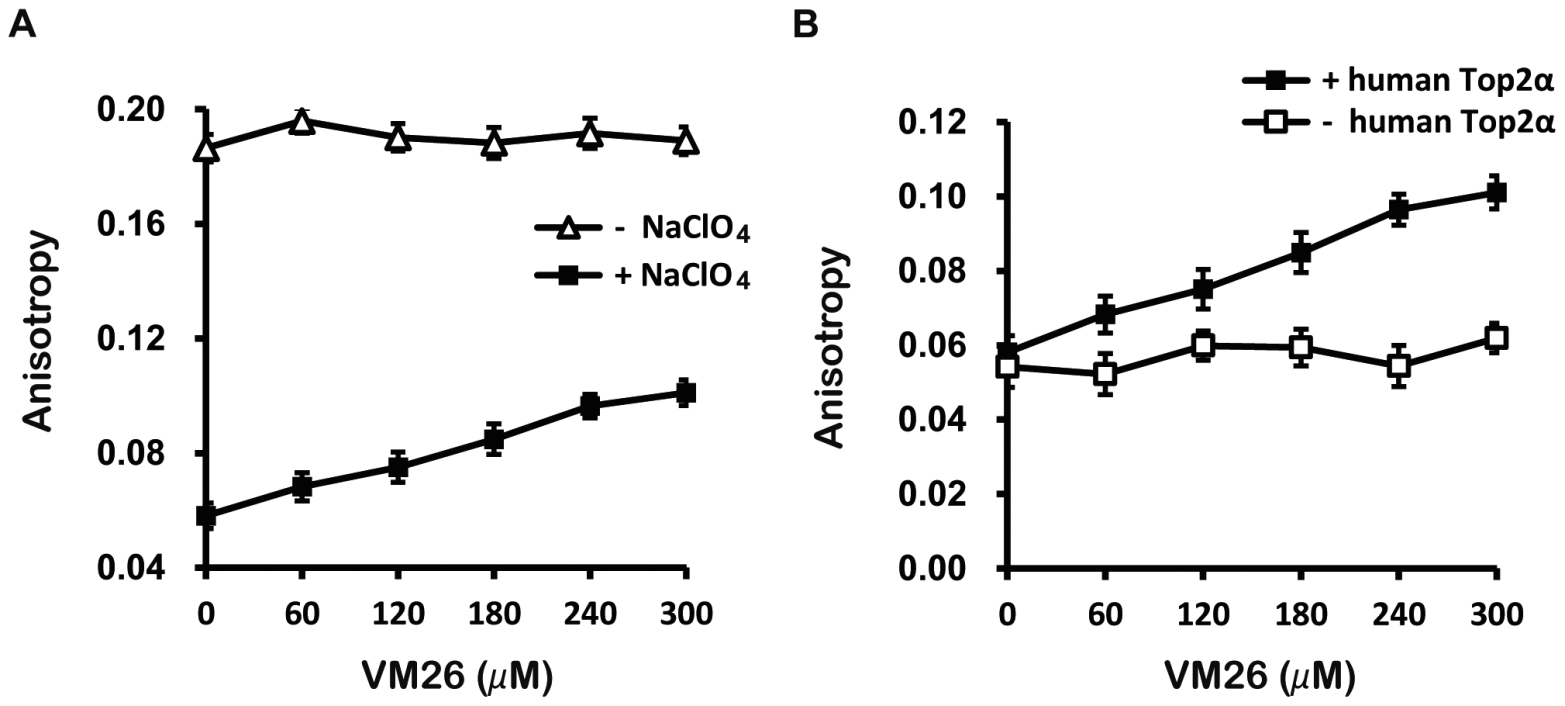
Dose-dependent anisotropy from VM26 after treatment with NaClO_4_. (A) Reactions of Alexa Fluor 488-labeled DNA with human Top2α were carried out with VM26 concentrations of 60, 120, 180, 240 and 300 µM. A dose-dependent increase in anisotropy with increasing VM26 concentrations was observed if the reactions were terminated with NaClO_4_ (solid square) but not without it (empty triangle). (B) The dose-dependent increase of anisotropy with increasing VM26 was observed when the reactions contained human Top2α (solid square) but not without it (empty square).

Using teniposide (VM26) as a model compound for our screening assay, we tested whether a number of commonly employed denaturing reagents including alkali and SDS can allow for detecting the enhanced stability of the Top2α-DNA complexes in the presence of an anti-cancer drug. Since alkali forms fine precipitates with Mg^++^ in the reaction buffer and SDS generates miniscule bubbles, both reagents create unacceptable artifacts for the microtiter-based fluorescence assays. However, we discovered that a strong chaotropic reagent NaClO_4_ meets our requirements as a dissociating reagent in the fluorescence assays. After the addition of NaClO_4_ to 250 mM to the binding reaction mixture, the stability of Top2α-DNA complexes as monitored by the fluorescence anisotropy shows a clear dose-dependent increase as VM26 concentrations varying from 0 to 300 µM (Fig 2A). Without adding NaClO_4_ the anisotropy remains the same regardless of the VM26 concentration. To further confirm that the VM26-induced anisotropy change is enzyme dependent, we performed the anisotropy measurement without topoisomerase in the otherwise identical reaction conditions. From the results shown in Fig 2B, we confirmed that drug dependent increase of anisotropy after treatment with NaClO_4_ was Top2α-mediated.

### Addition of NaClO_4_ abrogates Top2α binding to DNA, but retains the drug-stabilized enzyme-DNA ternary complexes

**Figure 3:**
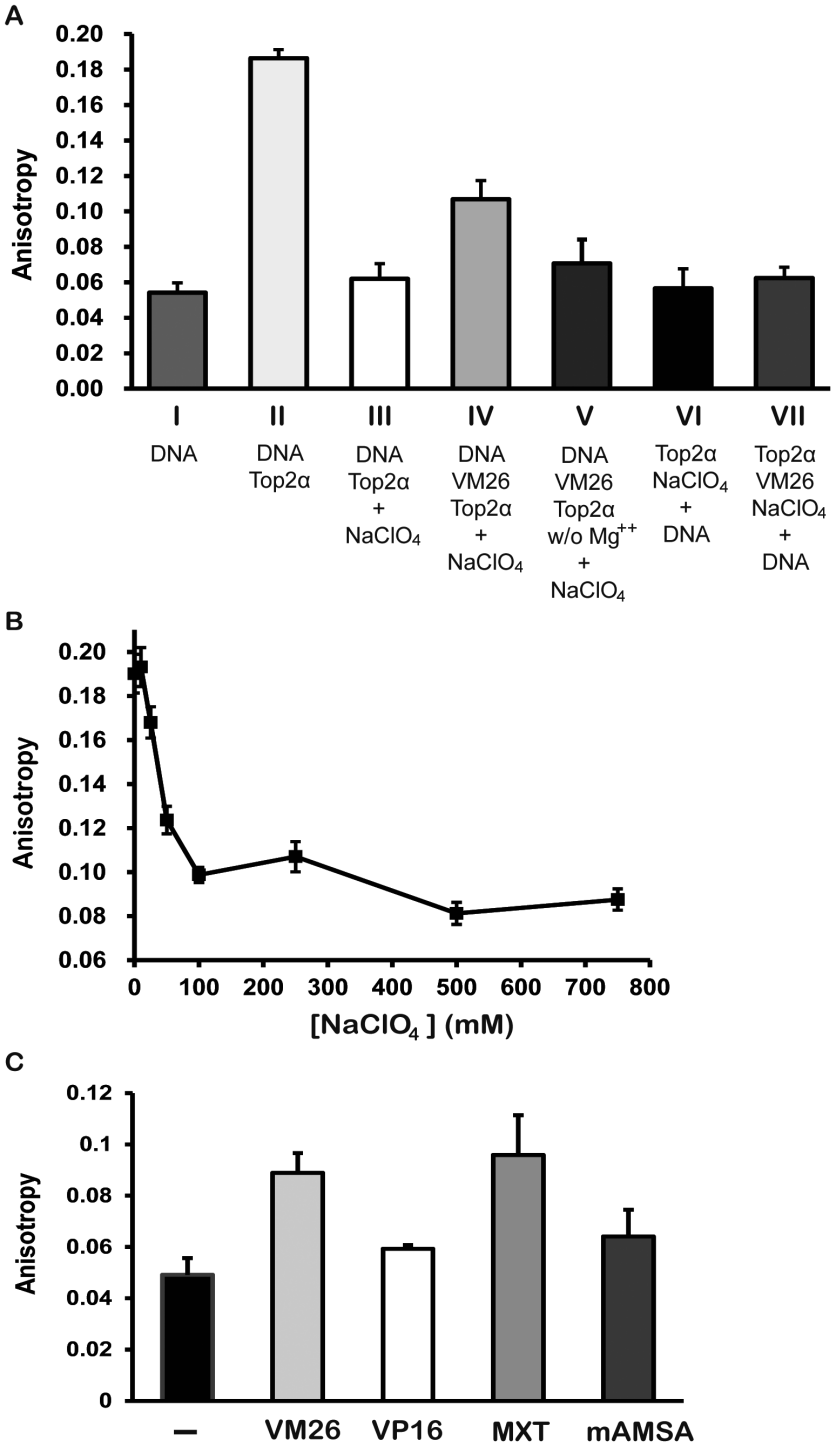
NaClO_4_ abrogates Top2α binding to DNA, but retains the drug-stabilized Top2α-DNA ternary complex. (A) The VM26-dependent increase in anisotropy was observed when the reaction was terminated with NaClO_4_ (Reaction IV vs Reaction III), and not when NaClO_4_ was added in the presence of Top2α before the addition of DNA, regardless of the addition of VM26 (Reactions VI and VII vs Reaction IV). Anisotropy observed in the controls was DNA only (Reaction I), DNA/Top2α without VM26 (Reaction II), and incubation without Mg^++^ (Reaction V). (B) Optimization of NaClO_4_ concentration for anisotropy assay. The reaction was performed in 50 µl Top2 reaction buffer with 300 µM VM26, 50 nM Alexa Fluor 488-labeled DNA and 250 nM human Top2α. The reaction mixture was incubated at 37°C for 30 minutes. Before measuring the anisotropy, NaClO_4_ was added into each reaction mixture to give a final concentration up to 750 mM. The maximal extent of dissociation was reached at a concentration of 100 mM or above. (C) Anisotropy observed with etoposide (VP16), mAMSA, mitoxantrone (MXT), and with teniposide (VM26) as positive controls, after treatment with 200 mM NaClO_4_. The concentrations of these known Top2α drugs used here were 300 µM for VP16 and VM26, and 15 µM for mAMSA and MXT.

In order to examine whether the increase of anisotropy after the addition of NaClO_4_ comes from the Top2α–bound DNA complexes, we tested the effects of altering the order of addition of key components in these reactions. In the absence of NaClO_4_, the anisotropy of DNA-enzyme complexes is 0.19 (Fig 3A-II), and the addition of NaClO_4_ reduces the anisotropy to 0.06 (Fig 3A-III), a value close to that of free oligonucleotide (Fig 3A-I). This result demonstrates that NaClO_4_ is capable of dissociating enzyme from DNA. While the formation of enzyme-DNA and teniposide (VM26) ternary complex results in NaClO_4_-resistant anisotropy (Fig 3A-IV), incubation without Mg^++^ (Fig 3A-V) or pre-incubation of the enzyme with NaClO_4_ prior to the addition of DNA, regardless of the presence of VM26, leads to a lower anisotropy (Fig 3A-VI and VII). This result suggests that NaClO_4_ is able to inactivate the enzyme and inhibit enzyme's ability to form DNA bound complexes. Since Mg^++^ is required for the enzymatic activities of topoisomerases, the requirement of Mg^++^ suggests that simple binding is not sufficient for the formation of NaClO_4_-resistant complex. Except for the VM26-stabilized ternary complexes, the treatment with NaClO_4_ in all other conditions reduces the anisotropy to a value similar to the free DNA substrate, confirming that NaClO_4_ is able to disrupt enzyme-DNA complexes without a Top2α-targeting agent. To demonstrate the concentration dependence of NaClO_4_ to allow for specifically detecting the VM26-stabilized enzyme-DNA complexes, we analyzed the anisotropy of binary complex after adding different amount of NaClO_4_ (Fig 3B). The maximal dissociation levels were reached at a NaClO_4_ concentration of 100 mM, with the IC_50_ at 40 mM, and there are little variations up to 750 mM. We have thus demonstrated that NaClO_4_ is an efficient agent to disrupt protein/DNA complexes with a broad concentration range between 100–750 mM, and it can be adapted for the use in HTS for Top2α-targeting agents.

To test that our anisotropy assays can be effective in identifying other known Top2α-targeting anti-cancer drugs besides teniposide (VM26), we measured anisotropy after NaClO_4_ treatment with etoposide (VP16), mAMSA, and mitoxantrone (Fig. 3C). Similar to that measured with teniposide, all three known cancer drugs produced anisotropy readouts above the controls.

### NaClO_4_ can promote the generation of nicked DNA in the drug-stabilized Top2α ternary complexes

**Figure 4:**
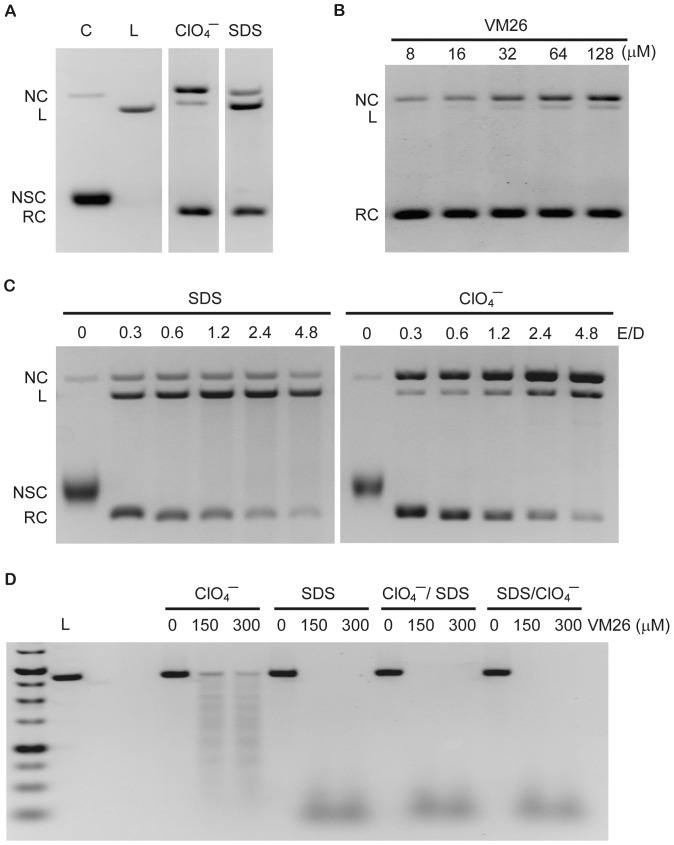
NaClO_4_ induces the formation of cleavage complexes with a preference of nicked DNA. (A) Reactions of human Top2α and pUC19 plasmid DNA were terminated with either NaClO_4_ or SDS. Addition of NaClO_4_ to 100 mM leads to the formation of more nicked DNA products while the addition of SDS results in a preference of linear ones. Reactions were carried out with 8.5 nM pUC19, 3.75 nM Top2α, and 100 µM VM26, terminated with the addition of either NaClO_4_ or SDS, the reaction products were treated with proteinase K, and analyzed by electrophoresis in 1.2% agarose gel containing 0.15 µg/ml ethidium bromide. (B) DNA cleavage assays were performed under similar conditions except with increasing concentrations of VM26 followed by addition of NaClO_4_. An increase in the nicked DNA cleavage products was observed with higher amounts of VM26. (C) Plasmid DNA cleavage assays were carried out using different enzyme to DNA ratios as indicated. Linear DNA products (full-length or sub-full length) from SDS treatment, and both nicked and linear DNA from NaClO_4_ treatment increase with higher enzyme to DNA ratios. (D) Reactions in the presence of various concentrations of VM26 contained linear pUC19 as substrate with an enzyme/DNA ratio of 20, followed by adding NaClO_4_ or SDS, or both sequentially, to stop the reactions. The size markers, and linear pUC19 were loaded in the leftmost two lanes. Positions of DNA were marked as NC (nicked circular), L (linear), NSC (negative supercoiled), and RC (relaxed).

The effects of NaClO_4_ in disrupting Top2α-bound DNA complexes were investigated with an oligonucleotide substrate monitored by fluorescence anisotropy assays. To further examine the potential mechanistic roles of NaClO_4_ in Top2α reactions, we decide to use plasmid DNA as the substrate which affords the application of agarose gel electrophoresis to examine the DNA structural changes. With a strong denaturant SDS to terminate reactions with plasmid DNA, the presence of an anti-cancer drug like teniposide (VM26) can entrap the cleavable complexes and increase the products of linearized and nicked DNA. We carried out experiments to investigate the DNA products generated from a reaction mixture containing Top2α-DNA-VM26 ternary complexes that were terminated with either SDS or NaClO_4_. To remove the proteins that were associated with DNA after the treatment with these dissociating reagents, the stopped reaction mixtures were treated with proteinase K before their analysis with agarose gel electrophoresis. The reaction products after being terminated with SDS showed the expected pattern of the appearance of linearized DNA predominantly, and some nicked species as well (Fig 4A). In an interesting contrast, the reaction products generated from NaClO_4_ treatment yielded more nicked DNA products and less linearized form. With NaClO_4_ to stop the reactions, the production of nicked and linearized forms increased in parallel upon increasing VM26 concentrations in these reactions (Fig 4B), demonstrating that both products were likely generated through VM26's action in entrapping cleavable complexes. Previous report already showed that VM26 promotes the formation of single stranded DNA nicks, in addition to double stranded breaks, depending on the concertedness of the cleavage from two protomers in Top2 dimer [21]. Both biochemical and structural biological data demonstrated that each protomer can bind a single drug molecule, the presence of which can affect the cleavage/religation mediated by the dimeric enzyme [22], [23]. The result that NaClO_4_ tends to generate less linearized cleavage products, and more nicked form, is possibly due to that it is a less potent denaturant than SDS and less efficient to induce the formation of cleavage complexes. One can thus expect to observe more linearized products with the presence of more enzyme and consequentially more cleavable complexes. We could show that by increasing the enzyme to DNA stoichiometry, the linearized DNA products increased concomitantly with the nicked form when the reactions were terminated with NaClO_4_ (Fig 4C). Under the same reaction conditions but terminated with SDS, the linearized products were further degraded to yield DNA fragments smaller than the unit length molecule. DNA cleavage and nicking induced by NaClO_4_ are not restricted to the circular DNA substrates. We could readily demonstrate DNA cleavage with linear substrate at higher enzyme/DNA ratios (Fig 4D). The degradation of DNA to generate sub-full length molecules from NaClO_4_ treatment is due to the close spacing between two nicks on juxtaposed strands and also some double strand cleavage under such conditions. However, under the same conditions the addition of SDS can induce more extensive DNA degradations, demonstrating that SDS is a stronger denaturant and more efficient in triggering Top2α cleavage reaction. Interestingly, when SDS and NaClO_4_ were added sequentially, SDS can promote the generation of smaller DNA products even when it was added after NaClO_4_. This result again suggests that NaClO_4_ does not completely disrupt enzyme/DNA/drug ternary complexes, still maintaining their sensitivity toward SDS in promoting more cleavage complexes.

### NaClO_4_-treated ternary complexes analyzed by ultracentrifugation in glycerol gradients

**Figure 5:**
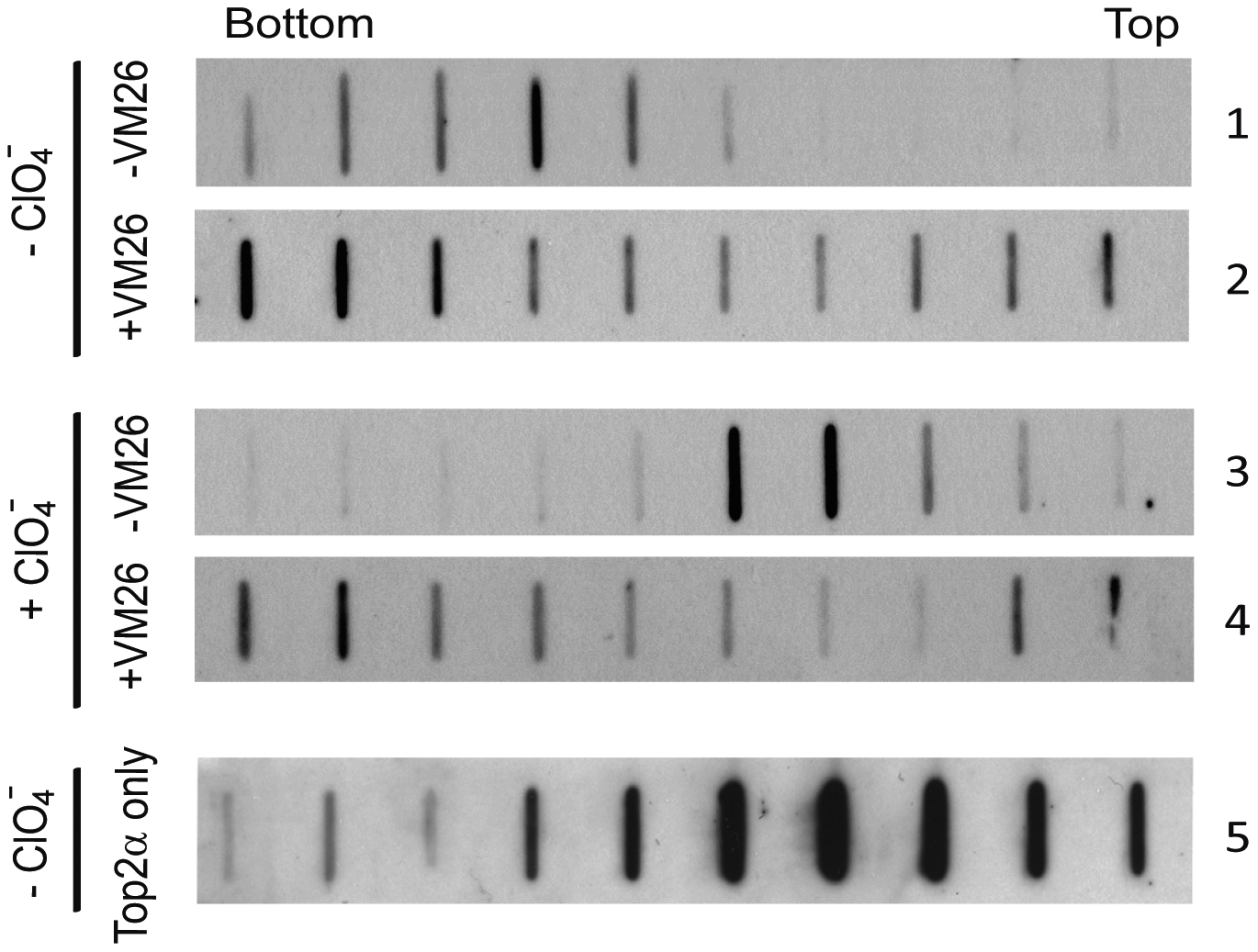
Glycerol gradient sedimentation analysis of topoisomerase/DNA complexes. The glycerol gradient solution is layered in a polyallomer tube starting from 60% to 30% glycerol. The reaction for Top2α/DNA complex formation was performed in 80 µl of a solution containing 100 µM VM26, 34 nM pUC19 and 3.75 nM human Top2α. The reaction mixture was loaded on top of a 30–60% glycerol gradient, and tube was topped off with mineral oil. After ultracentrifugation, the fractions were collected from the bottom of the tubes. The distribution of Top2α in each fraction was analyzed by slot blotting and immunodetection.

We applied ultracentrifugation in glycerol gradients to investigate the stability of Top2α-DNA complexes after the treatment of NaClO_4_, and the effect of an anti-cancer drug. Glycerol gradient centrifugation was often used to separate macromolecules based on their size and shape. We incubated Top2α with plasmid DNA either in the presence or absence of VM26, and applied it to the glycerol gradient directly or after the treatment with NaClO_4_. The fractions were collected after the centrifugation and the locations of Top2α were determined by western blots with antibody against Top2α. In the absence of VM26, Top2α sedimented more toward the bottom without NaClO_4_ treatment (Fig 5, lanes 1 vs 3). The presence of NaClO_4_ shifts Top2α to a lighter fraction, in a position corresponding to free enzyme (Fig 5, lanes 3 vs 5), thus confirming the ability of NaClO_4_ to disrupt enzyme-DNA complex in the absence of an anti-cancer drug. In the presence of VM26, treatment with NaClO_4_ did not grossly change the sedimentation of Top2α, suggesting that NaClO_4_ still maintains most of the ternary complexes (Fig 5, lanes 2 vs 4). Experiments from both fluorescence anisotropy and glycerol gradient sedimentation support the notion that NaClO_4_ treatment provides a means to differentiate the stability of Top2α-DNA complexes depending upon the presence of an anti-cancer drug, thus offering us an approach for automated screening assay and making it possible for the use in HTS platform.

### Development of assay conditions for HTS platform

**Figure 6:**
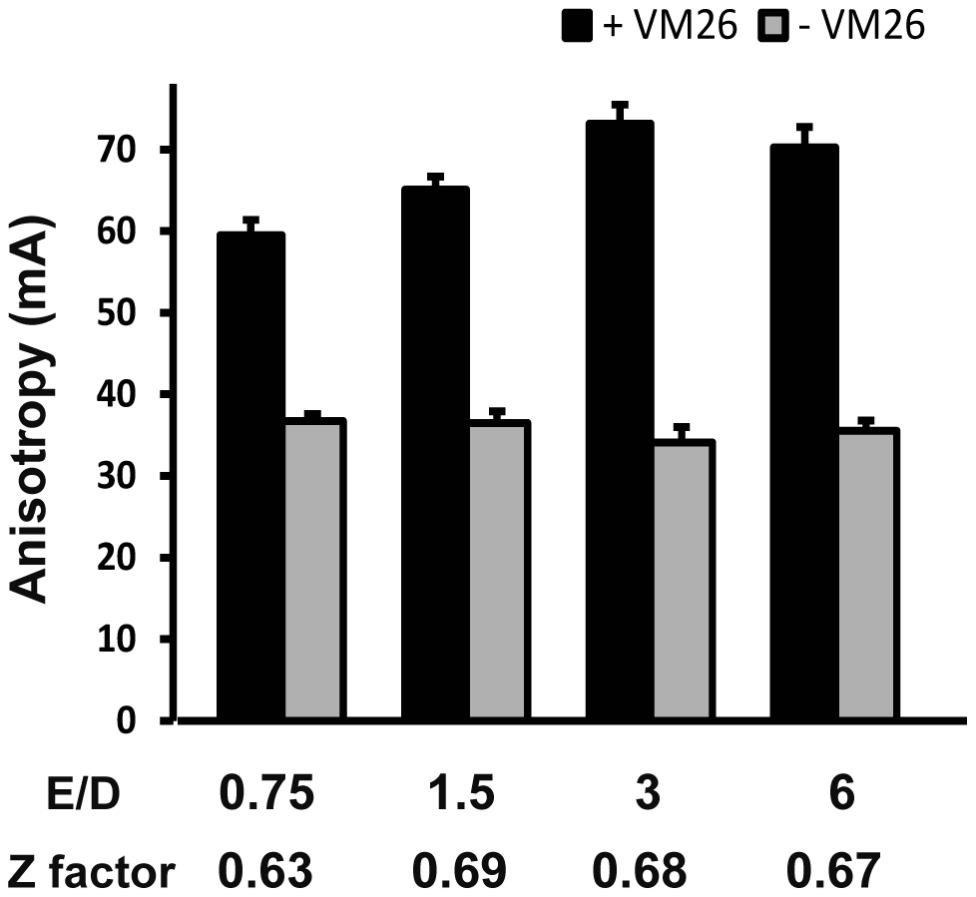
Assay development of high throughput screening. For development of HTS assay, we carried out reactions in 1536-well microtiter plate with various enzyme/DNA ratios in 4 µl of reaction buffer containing 10 mM Tris-HCl pH 7.9, 5 mM MgCl_2_, 50 mM NaCl, 0.1 mM EDTA, 0.5 mM ATP with or without 300 µM VM26. In the reaction mixture, the enzyme/DNA ratio started with 0.75 (150 nM enzyme, and 200 nM DNA) and increased by two-fold up to a ratio of 6. The reaction mixture was incubated at 37°C for 30 minutes. Before measuring the anisotropy, NaClO_4_ was added into each reaction mixture to give a final concentration of 100 mM. Z factor was calculated according to Zhang et al [24]. Anisotropy was measured in a unit of mA (milliAnisotropy).

Since our initial anisotropy assays were carried out in reaction mixtures of 50 µl, we would have to test whether these assay conditions were transferable to HTS platform carried out in 1536-well microtiter plates with a reaction volume of 4 µl in each well. With enzyme to DNA ratios between 0.75 and 6, we observed a consistent increase of anisotropy signals in reactions with teniposide (VM26) after NaClO_4_ treatment (Fig 6). A more important test is the evaluation of Z factor which computes the robustness of the signals based on both the measurements of signals and signal to noise ratios under different conditions. In this Z factor test, an HTS assay is feasible if Z factor is between 0.5 and 1, and the higher the Z factor is, the better the assay is [24]. Based on the data shown in Fig 6, we computed the Z factors, and they are all above 0.6 for the reactions in the range of enzyme/DNA ratios tested here. These results thus demonstrated that our screening conditions are adaptable to HTS platform.

### Confirmation of positive hits from HTS by biochemical assays

**Figure 7:**
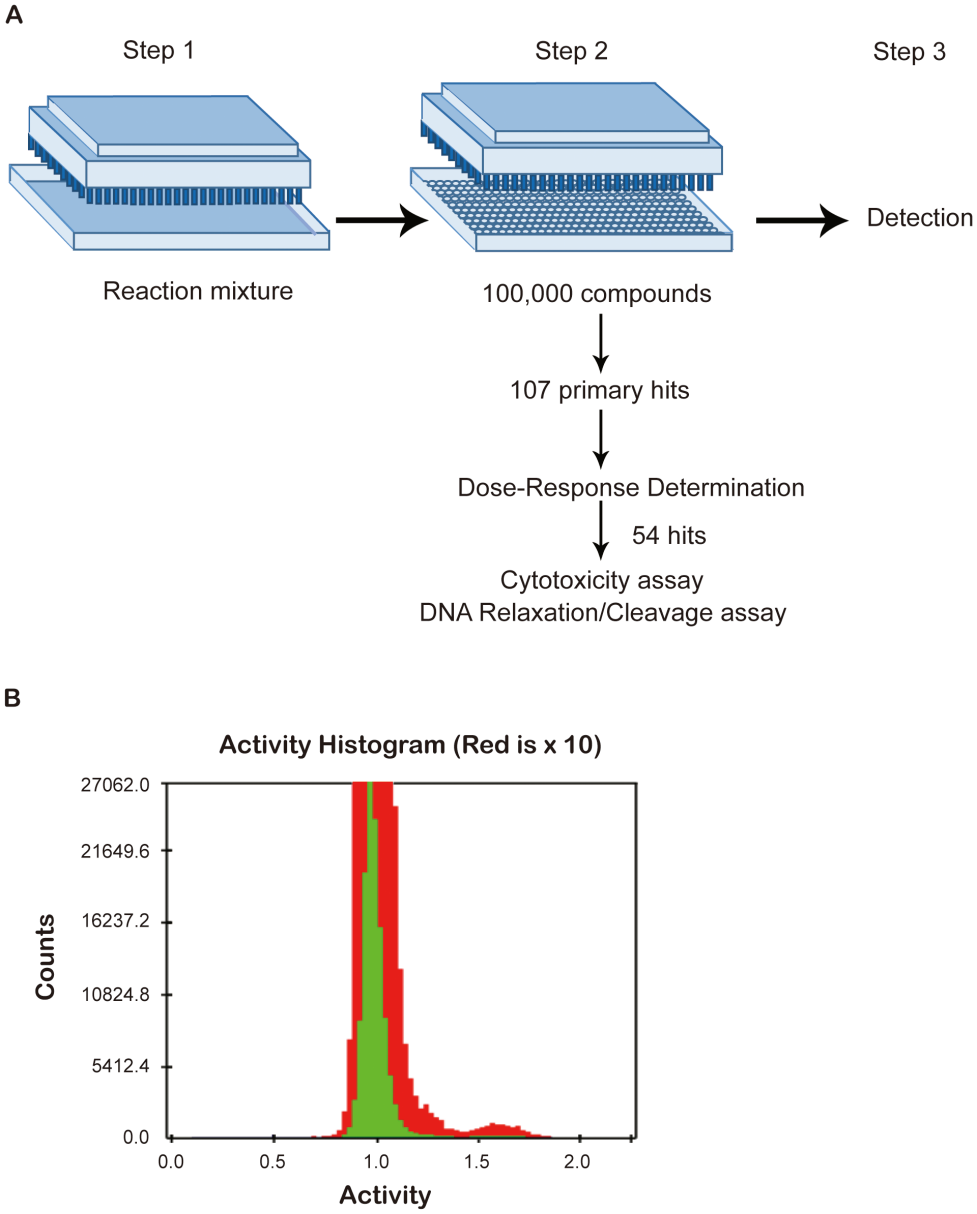
HTS process diagram and outcomes. (A) Diagrammatical representation of HTS procedure with 1536-well microtiter plates. The detailed screening protocol is described in Materials and Methods. (B) Distribution histogram of activities assayed. Activity is determined by taking the anisotropy ratio of the test compound over the mock controls. The ordinate is for the number of compounds screened. To highlight the bimodal distribution, red portion is for 10× compound counts of green portion.

**Figure 8:**
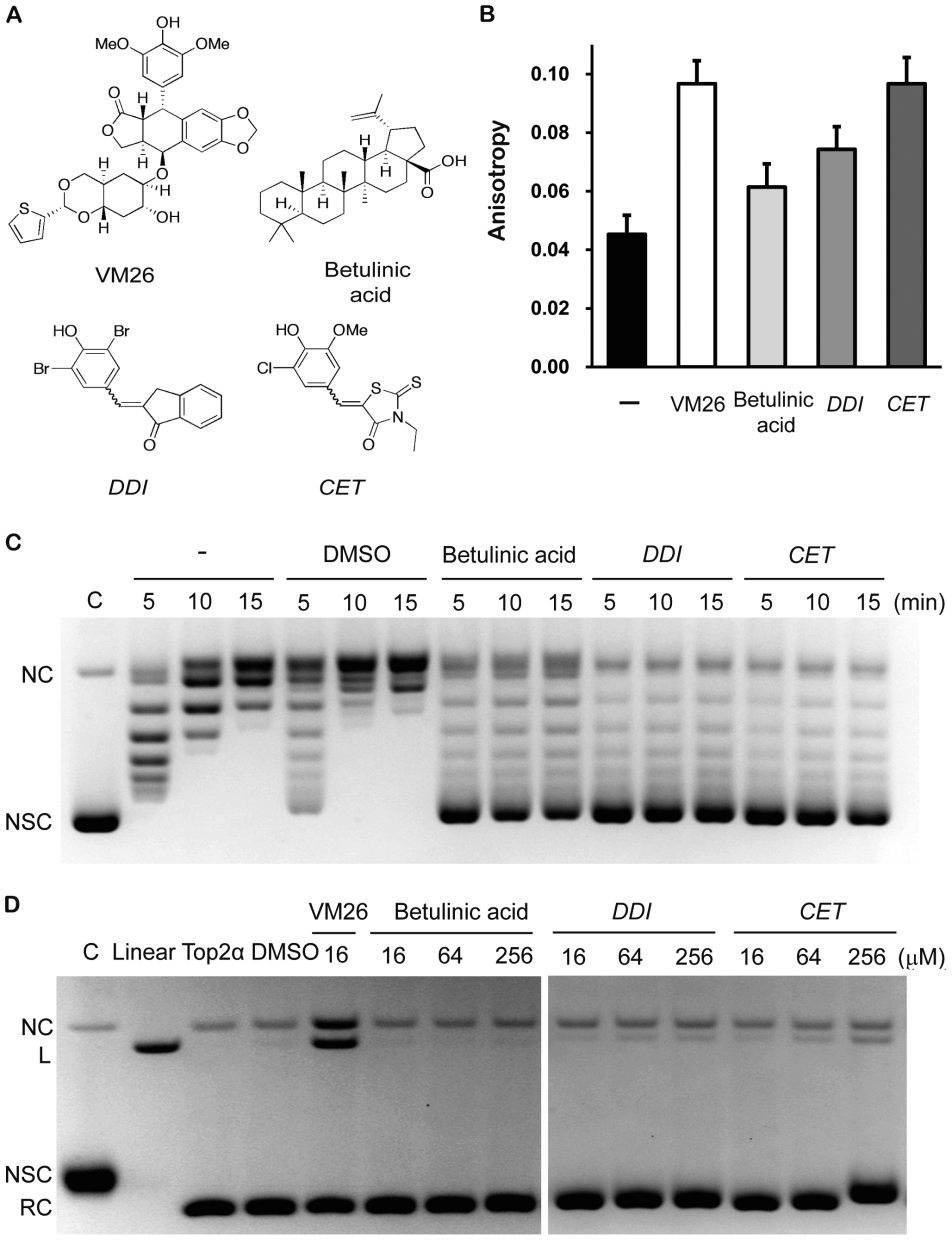
Confirmation of the positive hits. (A) Chemical structure of VM26 and three positive hits, betulinic acid, *DDI* and *CET*. (B) Anisotropy of enzyme/DNA/drug ternary complexes. The reaction was performed in 50 µl of reaction buffer with 50 nM Alexa Fluor 488-labeled DNA and 250 nM human Top2α. Negative control (filled) contained neither VM26 nor test compounds. Positive control (open) contained 300 µM VM26. Others (gray) contained 15 µM of test compounds. The reaction mixture was incubated at 37°C for 30 minutes. Before measuring the anisotropy, NaClO_4_ was added into the reaction mixture to give a final concentration of 200 mM. (C) Effects of positive hits on inhibition of DNA relaxation. The reaction contained 8.5 nM pUC19 and 0.125 nM human Top2α in 20 µl reaction buffer, and performed at 37°C for 5, 10 or 15 minutes. Solvent control contained 5% DMSO, and the other reactions contained 60 µM hit compound (DMSO was used as the solvent and its final concentration in each reaction was 5 %). (D) Effects of positive hits on DNA cleavage assay. The 20 µl reaction contained 8.5 nM pUC19 and 3.75 nM human Top2α with Top2α targeting compounds at the concentrations marked above each lane. Reaction products were analyzed by electrophoresis in a 1.2% agarose gel containing 0.15 µg/ml ethidium bromide. Positions of DNA were marked as NC (nicked circular), L (linear), NSC (negative supercoiled), and RC (relaxed).

With the assay conditions developed here, we carried out HTS from 100,000 compounds in our chemical library using a concentration of 12.5 µM of test compound in the reaction mixtures. The flow chart of the screening process is shown diagrammatically in Fig. 7A. The distribution histogram of anisotropy data revealed an interesting bi-modal distribution (Fig. 7B). The vast majority of the compounds gave rise to an average anisotropy value of about the same as the background controls (thus with a ratio of 1.0 in the figure). Interestingly, there is a separate peak of anisotropy group that is about 60% above the background value (ratio of 1.6). This is more evident in the red portion of the graph, which is a 10X-expansion of the ordinate of green portion (Fig. 7B). Since there is a clear and distinct separation between the bulk and the outlying group, we select for the compounds with anisotropy ratio greater than 1.4 as potential leads. This group of compounds, 107 in total, went through a second round of screening by testing the anisotropy readouts at 8 different concentrations of each candidate compound using a series of two-fold dilutions from 12.5 µM downward. Only the compounds resulting in 20% increases in detected signals over the negative controls and showing dose-dependent responses at two or more points were defined as hits. Based on these criteria, a total 54 hits were selected. To evaluate the validity of our screening platform, we selected a few compounds from these hits for further evaluations with biochemical analysis. Among the first-round positive hits, there is a known Top2-targeting inhibitor, betulinic acid [25]. We chose betulinic acid and two previously uncharacterized compounds from the second-round positive hits, 2-(3,5-dibromo-4-hydroxybenzylidene)-2,3-dihydro-1*H*-inden-1-one (*DDI*), and 5-(3-chloro-4-hydroxy-5-methoxybenzylidene)-3-ethyl-2-thioxothiazolidin-4-one (*CET*), to investigate their effects on Top2α reactions. These compounds have diverse chemical structures when compared with VM26 (Fig 8A), suggesting that the screening approach has a potential to pick up novel structures.

We re-examined the anisotropy measurements under the bulk platform and the results showed all three hits had considerably higher anisotropy than the controls, albeit they have different increments (Fig 8B). These results again reaffirm that the HTS platform using microtiter plates can yield reliable anisotropy readouts and the hits can be further evaluated.

We examined the effects of these hits on the biochemical properties of Top2α. In the presence of 60 µM of the candidate compounds, the supercoil relaxation kinetics were examined and analyzed by agarose gel electrophoresis (Fig 8C). While Top2α at a concentration of 0.125 nM can readily remove the plasmid supercoiling in 10 min of reactions either without or with DMSO (vehicle used to dissolve the candidate chemicals), the presence of 60 µM of all three test compounds effectively inhibit most of the catalytic activities of Top2α even after 15 min of incubation. Another hallmark reaction we tested was the promotion of strand cleavage by Top2α. We incubated plasmid DNA with 3.75 nM of Top2α and various amounts of the test compounds from 16 to 256 µM, and terminated the reactions with SDS to entrap the cleavable complexes. The reaction products were treated with protease and analyzed by agarose gel electrophoresis in the presence of ethidium to facilitate the detection of nicked and linearized cleavage products (Fig 8D). While teniposide (VM26) can promote DNA cleavage products, betulinic acid has minimal effects on DNA cleavage, a result consistent with an earlier finding that it is a catalytic inhibitor and not a Top2α poison [25]. Interestingly, both hits *DDI* and *CET* can slightly enhance the cleavage products, as evidenced by a small yet detectable increase in nicked and linearized DNA products. Therefore, it is possible that these two compounds may have dual roles in both as a catalytic inhibitor and a poison. Our screening platform may produce new types of potential anti-cancer agents targeting Top2α.

**Figure 9:**
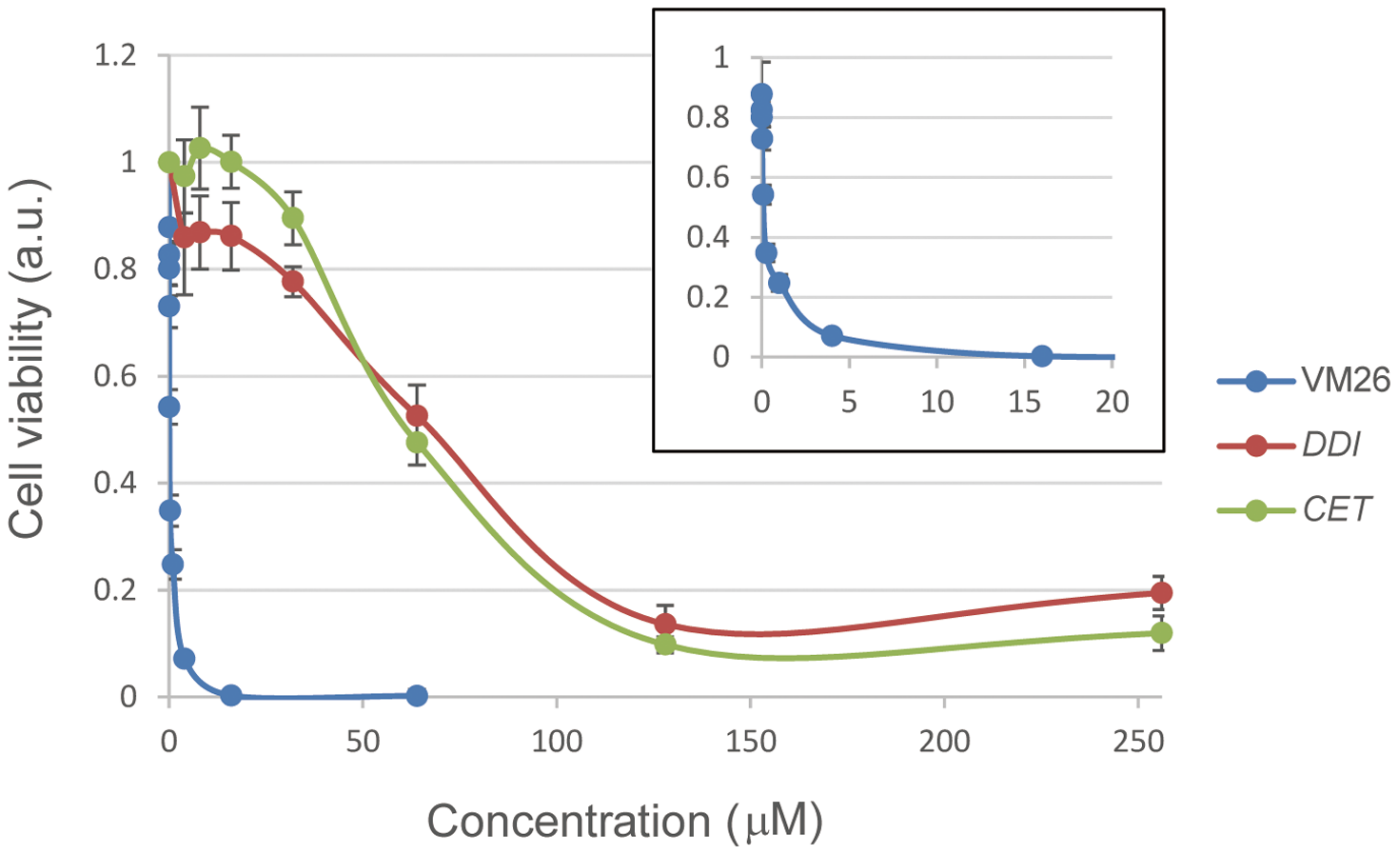
Cytotoxicity of the positive hits. HCT116 cells were treated with *DDI*, *CET*, and teniposide (VM26) at various concentrations, and their cytotoxicity was determined by MTS assays. The cytotoxicity toward teniposide is highlighted in the *insert*.

While the biochemical and biophysical data confirmed that the compounds from the HTS can target Top2α and affect their enzymatic properties, it is important to demonstrate that they are cytotoxic toward cancer cells and thus have the potential to be developed as anti-cancer drugs. We measured the cytotoxicity of *DDI* and *CET*, and compared with that of teniposide (VM26) using human colorectal cancer cell lines HCT116 (Fig. 9). The results demonstrated that *DDI* and *CET* have IC_50_ about 60 µM, and are thus able to kill cancer cells, albeit significantly less potent than the known anti-cancer drug teniposide which has a much lower IC_50_ in the sub-micromolar range (insert of Fig. 9).

## Discussion

DNA topoisomerases are essential enzymes with critical functions in modulating structures of DNA and chromosomes during replication, segregation, transcription, recombination, and repair. They also serve as intracellular targets for a number of clinically important antibiotics [26], [27], and anti-cancer drugs [12], [28]–[30]. Because of the prominent role of topoisomerases as the targets for chemotherapy and an ever-increasing need for new therapeutic agents to combat the rising drug-resistance, one would anticipate intensive efforts in screening for new drugs targeting this group of enzymes. However, these efforts are somewhat thwarted by the cumbersome nature of enzymatic assays available. Most of the biochemical assays were developed for monitoring the changes in DNA topology resulting from the enzymatic actions, and they involved the use of agarose gel electrophoresis which was time consuming, labor intensive, and not readily amenable to high volume screening protocols. Indeed there are a number of screening approaches developed recently by measuring either fluorescence or fluorescence anisotropy, and they can be adopted for high throughput platform [15]–[18]. All of these methods involve the use of covalently closed circular DNA as the substrate or part of the substrates used in the optical assays. In screening for the gyrase inhibitors, a fluorescent dye PicoGreen was used in the differential binding to the supercoiled DNA products [18]. Two methods shared the similar general approach of using the differential binding of a fluorophore-tagged oligonucleotide to supercoiled/relaxed DNA in the formation of triplex structure [31], [32]. A FRET system assaying for topoisomerase activities is designed with the donor/acceptor pair located in the apices across a cruciform that is sensitive to the plasmid supercoiling [17]. All three methods can be applied to HTS platform. Recently, HTS screening has been conducted with the differential triplex formation methods for human Top2α catalytic inhibitors [15], and for inhibitors of bacterial gyrase and Top VI [16], as well as with cruciform extrusion method for gyrase inhibitors [17].

We endeavor to develop a simple and facile screening based on the measurement of fluorescence anisotropy to discover effective anti-tumor drugs targeting Top2α. One key feature of this screening strategy is that it is based on a fluorophore-labeled oligonucleotide duplex which is simple to prepare for sufficient amounts in high throughput platform. The second feature is that it monitors the stable binding of Top2α to DNA, thus allowing for the potential to discover both poison type drugs that promote the formation of cleavable complexes and the catalytic inhibitor that may involve slow dissociation and turnover of the enzyme. We choose as the substrate a 29-bp oligonucleotide duplex with an Alexa Fluor 488 at its 3′-end, and its sequence contains a binding/cleavage site for eukaryotic Top2 [33]–[35]. The DNA substrate with such a sequence has been extensively used for the investigation of Top2/DNA interactions [36]–[38]. We anticipate that binding of Top2α to this sequence can slow down the rotational diffusion and increase the fluorescence anisotropy of DNA substrate.

To realize the second feature, we will need a dissociating reagent that can maintain the enzyme/DNA ternary complex with a Top2α targeting agent and disrupt Top2α binding to DNA without it. The choice is not as apparent as it seems. The obvious ones including strong denaturants SDS and NaOH are not suitable for our HTS methods. The addition of SDS tends to generate miniscule bubbles and the hydroxide reacts with Mg^++^ in the reaction buffers to produce fine precipitates, both of which interfere with fluorescence measurements. We discovered that a chaotropic agent, NaClO_4_, worked well for our screening purpose. Using teniposide (VM26) or etoposide (VP16) as model compounds, including NaClO_4_ in our assays can generate a reproducible signal for fluorescence anisotropy readouts. Based on the Z-factor criterion this new screening method is able to generate robust signals for HTS platform. The addition of NaClO_4_ can disrupt Top2α/DNA binary complex based on the results from both fluorescence anisotropy and glycerol gradient sedimentation. Interestingly, fluorescence anisotropy and glycerol gradient sedimentation also demonstrate that NaClO_4_ allows for the complex formation between DNA/enzyme in the presence of a Top2α-targeting drug. Experiments with agarose gel electrophoresis further show that NaClO_4_ promotes the cleavage complex under such conditions. However, there are differences between the cleavage complex induced by NaClO_4_ when compared with those triggered by SDS. The most apparent one is that NaClO_4_ tends to generate more nicked products while SDS generates more linearized ones. It is possible that NaClO_4_ is a less potent denaturant in comparison with SDS, thus leaving an enzyme/DNA complex capable of undergoing partial reversion from the double-stranded cleavage state to a single-stranded, nicked state. Early work also showed that the reversion of the cleavage complex by the treatment of heat or high salt plus EDTA is incomplete and the subsequent addition of a denaturant results in predominantly nicked DNA products [39]. It is evident from the reactions with Top2 under unfavorable conditions including the use of divalent cations other than Mg^++^ or acidic pH that the reversion of the double stranded breaks in the cleavage complex can be incomplete and unconcerted [40], [41]. The notion that NaClO_4_ is only capable of incomplete disruption of the cleavage complex is additionally supported by experiments testing the effects of the order of addition of reagents in terminating the Top2α reactions. While the addition of SDS results in more extensive DNA cleavage than NaClO_4_, it is interesting that adding SDS after NaClO_4_ still generates a similar extent of cleavage as SDS alone or SDS added before NaClO_4_ (see Fig. 4D). This result suggests that in the presence of NaClO_4_ the ternary complex of enzyme/DNA and Top2α-drug can maintain a conformation sensitive to the effect of SDS in promoting more extensive DNA cleavage. These unique features of NaClO_4_ may therefore be able to provide us a HTS platform in screening for new types of Top2α-targeting agents. The assay protocol reported here may also be adapted for screening for agents specific to other DNA topoisomerases.

We have applied the HTS protocol described here in searching for Top2α-targeting agents in a chemical library of 100,000 compounds and from which we obtained 54 positive hits. The signals from these hits were robust and reproducible over a range of concentrations of test compounds used in the assays. However, the selective criterion we applied was very stringent, and it was therefore possible that some of the potential positive hits may have been overlooked in exchange for minimizing false positives. As an initial characterization for these positive hits, we have selected three from them for further analysis. They include one previously known compound (betulinic acid) and two not known to target Top2α. Both fluorescence anisotropy measured under the bulk conditions and the relaxation and cleavage assays using agarose gel electrophoresis demonstrated their effects on Top2α reactions. It is interesting to note that while betulinic acid is an effective catalytic inhibitor as reported previously [25], the other two compounds characterized here showed both inhibitory and poison-type activities. It is therefore possible that our screening platform may uncover diverse types of Top2α-targeting agents and may serve as a new direction for the search of future drugs targeting Top2 and other DNA topoisomerases.
